# Experimental increase in baseline corticosterone level reduces oxidative damage and enhances innate immune response

**DOI:** 10.1371/journal.pone.0192701

**Published:** 2018-02-12

**Authors:** Csongor I. Vágási, Laura Pătraș, Péter L. Pap, Orsolya Vincze, Cosmin Mureșan, József Németh, Ádám Z. Lendvai

**Affiliations:** 1 Evolutionary Ecology Group, Hungarian Department of Biology and Ecology, Babeş-Bolyai University, Cluj Napoca, Romania; 2 Behavioural Ecology Research Group, Department of Evolutionary Zoology, University of Debrecen, Debrecen, Hungary; 3 Department of Molecular Biology and Biotechnology, Babeş-Bolyai University, Cluj Napoca, Romania; 4 Emergency Hospital, University of Agricultural Sciences and Veterinary Medicine, Cluj Napoca, Romania; 5 Department of Pharmacology and Pharmacotherapy, University of Debrecen, Debrecen, Hungary; Universidad de Granada, SPAIN

## Abstract

Glucocorticoid (GC) hormones are significant regulators of homeostasis. The physiological effects of GCs critically depend on the time of exposure (short vs. long) as well as on their circulating levels (baseline vs. stress-induced). Previous experiments, in which chronic and high elevation of GC levels was induced, indicate that GCs impair both the activity of the immune system and the oxidative balance. Nonetheless, our knowledge on how mildly elevated GC levels, a situation much more common in nature, might influence homeostasis is limited. Therefore, we studied whether an increase in GC level within the baseline range suppresses or enhances condition (body mass, hematocrit and coccidian infestation) and physiological state (humoral innate immune system activity and oxidative balance). We implanted captive house sparrows *Passer domesticus* with either 60 days release corticosterone (CORT) or control pellets. CORT-treated birds had elevated baseline CORT levels one week after the implantation, but following this CORT returned to its pre-treatment level and the experimental groups had similar CORT levels one and two months following the implantation. The mass of tail feathers grown during the initial phase of treatment was smaller in treated than in control birds. CORT implantation had a transient negative effect on body mass and hematocrit, but both of these traits resumed the pre-treatment values by one month post-treatment. CORT treatment lowered oxidative damage to lipids (malondialdehyde) and enhanced constitutive innate immunity at one week and one month post-implantation. Our findings suggest that a relatively short-term (i.e. few days) elevation of baseline CORT might have a positive and stimulatory effect on animal physiology.

## Introduction

The proper maintenance of homeostasis is central to all living organisms. Glucocorticoid (GC) hormones are released via activation of the hypothalamic–pituitary–adrenal (HPA) axis and are prominent regulators of homeostasis [1]. GCs orchestrate a vast repertoire of behavioral and physiological adjustments in response to changes in the external or internal environment. However, the effects of GCs might vary depending on the length of exposure and the extent of GC level elevation [2,3]. Long-term (days/weeks/months) and substantial increases in GCs were found to be detrimental to organisms and to impair performance, while short-term (minutes/hours/days) and mild (i.e. within baseline range) elevations in GC levels may have positive effects, either via preparation or hormesis [4,5]. Nonetheless, such effects need further scientific attention given that few experimental studies manipulated GC levels within the baseline hormone range (but see [6–8]; reviewed by [9]) and assessed the consequences of more natural GC levels on fitness or physiology.

It is increasingly recognized that GC-mediated stress response maintains homeostasis by regulating components of the physiological regulatory network [10], including two main domains of the latter: immune system and oxidative balance. GCs were traditionally considered to inevitably exert a suppressive effect on the immune function [11]. However, an unconditional GC-induced immunosuppression would not be adaptive as the proper functioning of the immune system is essential for sustaining homeostasis, as well as for recovery from stress [11–13]. Several recent studies have demonstrated that short-term and mild increases in GC levels enhance rather than suppress the immune function ([14]; reviewed by [11,12,15]). The current view is that short-term and mild elevation in GC levels can be beneficial either by priming the immune system or via a hormetic effect, whereas prolonged high levels of GCs are more detrimental by causing an allostatic overload that ultimately results in a down-regulation of survival-enhancing functions, such as immunity [4,12,13,15].

Oxidative state is another physiological axis with strong effects on homeostasis. Oxidative stress is the state of imbalance between pro-oxidants produced by normal cellular respiration and the antioxidant defense system in favor of the former, and it implies the oxidative damage of vital cellular components (lipids, proteins and DNA) [16]. Oxidative stress is functionally linked to both GCs and immune function [17,18]. Accumulating evidence suggests that GCs cause oxidative stress, disrupt cellular integrity and ultimately contribute to the ageing process (reviewed by [17,19]). However, GCs can also stimulate the expression of genes that encode antioxidant enzymes and consequently reduce oxidative stress [17]. The majority of experimental studies conducted to date induced a short-term (i.e. few days) elevation in GC levels well above the natural baseline (or even the peak) levels [17]. The effect of such extreme GC levels might contrastingly differ from that of chronic stress [20,21]. For instance, it was demonstrated that the GC-induced oxidative stress is mediated by the low-affinity GC receptors that are only activated at high GC concentrations [22]. The effect of chronic but mild increase in GCs on redox state are much less studied [17]. Moreover, the vast majority of studies that investigated GC-induced oxidative stress are either *in vitro* (organelles and cell cultures) or *in vivo* that involved laboratory model organisms with often limited applicability to free-living animals [17,23,24]. Only a handful of studies assessed the effect of GC administration on redox state in free-living species either in their natural environment or in captivity (reviewed by [17]).

Here we studied the effect of a within baseline range and long-term elevation of GC level on both condition (body mass, hematocrit and infestation) and physiological state (immune system and oxidative balance). We experimentally increased corticosterone level (CORT; the main GC in birds) using 60 days release pellets implanted in wild-caught house sparrows *Passer domesticus*. We followed the effects of treatment over two months at multiple levels (as suggested by [25]); (1) cellular level: glutathione (an intra-cellular non-enzymatic antioxidant) and phospholipid peroxidation; (2) individual (physiological) level: hematocrit, constitutive innate immune activity (natural antibodies and the complement system), plasma non-enzymatic antioxidants (total antioxidant capacity and uric acid), body condition and coccidian (intestinal parasite) infestation. We predicted that the exogenous hormone treatment will (1) induce a chronic increase in circulating CORT within the baseline range (level B; *sensu* [26]); (2) body mass and hematocrit will decline according to the duration of manipulation [27]; and (3) on the short-term (i.e. few days), treatment will enhance innate immunity and reduce oxidative stress [12,17], but a long-term (i.e. weeks/months) chronic elevation of CORT will suppress the immune system, exacerbate coccidian infestation and induce oxidative stress [12,17,27].

## Materials and methods

### Study animals and general procedures

Adult male house sparrows (*n* = 42) were caught on two cattle farms (near Cojocna and Copăceni villages, Transylvania, Central Romania) between 2 and 5 August 2011. Shortly after capture, birds were transported to the campus of the Babeş-Bolyai University, Cluj Napoca (46°46'N, 23°33'E, approximately 30 min travel) and were housed in two indoor aviaries (4 × 3.5 × 4 m each) in two equally-sized flocks. Aviary conditions are detailed in the Ethics Statement below. Photoperiod was set to be identical to the natural light:dark regime throughout the experiment. Birds were left to acclimate until they finished their complete post-breeding molt (i.e. till early November), consequently all individuals experienced identical captive conditions for over three months prior to treatment. The experiment started with CORT pellet implantation (see below) on 18 November 2011 and lasted until 26 January 2012.

### Study timeline and CORT treatment

The CORT implantation was carried out on 18 November 2011 (day 0). Prior to implantation, we took pre-treatment biometry measurements (body mass with Pesola spring balance ± 0.1 g; tarsus length with digital caliper ± 0.01 mm) and drawn a blood sample (100–150 μL) into heparinized capillaries from the brachial vein (*t*_0_ sampling session) to measure pre-treatment immune and oxidative state markers. The reason why we could not measure baseline CORT at *t*_0_ is that all birds were implanted on the same day (see below), and it was impossible to capture all birds and take blood samples from all of them within 3 min. Therefore CORT was not measured from the *t*_0_ samples, but the immune and oxidative stress parameters that are less responsive to short-term acute stress were analyzed from the *t*_0_. Blood samples were stored in a cooling box at ~4°C for no more than 2 h until being centrifuged for 5 min at 6,200 *g*. Plasma fractions were stored at –50° C until subsequent laboratory analyses (6 months at most).

Birds were randomly allocated to either of the two experimental groups, control or CORT-treated, and both aviaries had equal number of birds from both groups. CORT pellets (0.5 cm diameter, biodegradable carrier-binder; Innovative Research of America, Florida) contained a total dose of 2 mg CORT for an estimated 60 days release (i.e., 1.13 ng CORT / g body mass / day, computed with 29.5 g, the average mass of birds at pre-treatment). Control pellets were similar in size but without hormone content. Pellets were implanted subcutaneously through a small incision on the back, which was closed with sterile surgical thread once the pellet was inserted.

The two aviaries were only visually separated, therefore we left 3–5 days gaps between sampling of the two flocks to avoid the confounding effect of stress potentially caused by sampling the neighboring aviary. To follow the effect of treatment, birds were captured, measured and a blood sample was collected on day 5 and 10 (clumped as first post-treatment, *t*_1_, sampling session), day 30 and 33 (clumped as second post-treatment, *t*_2_ sampling session), and day 59 and 62 (clumped as third post-treatment, *t*_3_ sampling session). For each of these post-treatment sessions, the first date is for one aviary, while the second for the other (the order was randomized). On each occasion, several persons simultaneously took blood samples from the birds. Samples that were taken within 3 min (measured from the time when the first person entered the aviary) were used to measure baseline CORT. The rest of the birds in the same flock were also sampled, but their blood samples were used for hematocrit, immune and oxidative parameter quantifications. Sample sizes for CORT and physiology, respectively, were as follows: *t*_1_: 11 and 27; *t*_2_: 13 and 26; *t*_3_: 12 and 26. In order to measure the effect of CORT treatment on feather quality [28], we plucked the two innermost rectrices prior to the implantation. The fully grown replacement feathers were plucked at *t*_3_ and we measured their dry mass using an analytical balance (± 0.1 mg).

### Plasma CORT measurements

Plasma CORT concentrations were measured by direct radioimmunoassay (RIA) following extraction using 3 mL of diethyl-ether [29]. The extraction was reconstituted with phosphate-buffered saline (PBS). We used a commercial antiserum, raised in rabbits against CORT-3-(O-carboxymethyl) oxime bovine serum albumin conjugate (Sigma-Aldrich, St. Louis, MO; product number: C8784). The reconstituted extracts were incubated for 48 h at 4°C with 100 μL of [3H]CORT (Perkin Elmer, product number: NET399250UC) and antiserum (diluted to 1:8 after reconstitution as per the manufacturer’s instructions). The total volume of the assay was 1 mL. The radioactively labelled CORT had an activity of cca. 10K dpm. Bound and free CORT were separated by adding 100 μL dextran-coated charcoal (separation suspension: 10 g charcoal, 1 g dextran, 0.2 g commercial fat-free milk powder in 100 mL distilled water). After centrifugation, 800 μL of the bound fraction was added to 6 mL of scintillation cocktail (Optima Gold, Perkin Elmer) and counted in a liquid scintillation counter (Tri-carb 2800TR, Perkin Elmer). The minimum detectable level of CORT was 3.90 pg/tube and none of the samples fell below this limit. Mean recovery was 73.8% and intra-assay coefficient of variation was 7.4% based on 8 replicates. All samples were run in a single assay.

### Oxidative physiology markers

We assayed four markers of oxidative physiology, three indicating antioxidant potential (total antioxidant status, TAS; uric acid, UA; total glutathione, tGSH) and one showing the level of lipid peroxidation (malondialdehyde, MDA). Detailed protocol description can be found elsewhere [30].

TAS is a composite measure of antioxidant capacity, expressing the cumulative ability of all non-enzymatic antioxidants found in plasma, such as vitamins, sulfhydryl groups of proteins and uric acid, to combat a simulated free radical insult. TAS was measured colorimetrically, from 5 μL of plasma, using the TAS kit (Cayman Chemical, Ann Arbor, MI). This assay relies on the ability of antioxidants in the plasma to inhibit the formation of ABTS^+^ from oxidation of ABTS (2.2′-azino-di-(3-ethylbenz-thiazoline sulfonate)) by metmyoglobin. An antioxidant of known concentration (Trolox) was used as a standard for the calculation of antioxidant levels in the samples. Values of TAS are expressed as mM Trolox equivalents. Repeatability of a sub-sample measured twice was moderate but significant (intraclass correlation coefficient, ICC = 0.54, 95% CI = 0.034–0.832, *F*_12,13_ = 3.37, *P* = 0.019). Since uric acid (UA) is a component of TAS as well as a product of amino acid catabolism, we controlled TAS for UA levels by OLS regression and calculated residual TAS (as suggested by [31]). Results about residual TAS were highly similar to that of raw TAS and therefore were omitted throughout.

UA concentration of plasma was determined spectrophotometrically from 5 μL of plasma using the uricase/peroxidase method (Uric Acid liquicolor kit, Human, Wiesbaden, Germany). Results are given as mg/dL plasma. Repeatability of duplicate measures was very high (ICC = 0.99, 95% CI = 0.965–0.995, *F*_16,17_ = 15.3, *P* < 0.001).

Glutathione is the most significant intracellular, endogenous, non-enzymatic antioxidant [32]. tGSH concentration was assayed by means of a commercial kit (Sigma-Aldrich, St. Louis, MO; according to [32] and [33] with modifications [30]). Results are given in nmol/mg of red blood cells. The repeatability of the sub-sample measured twice was high (ICC = 0.82, 95% CI = 0.53–0.94, *F*_13,14_ = 9.88, *P* < 0.001).

MDA is a carbonyl compound that results from the peroxidative degeneration of membrane polyunsaturated fatty acids by reactive oxygen species, and thus it is a widely used marker of oxidative stress [34]. Briefly, MDA concentration was determined from 10 μL of plasma by High Performance Liquid Chromatography (HPLC) on a HPLC SUPELCOSIL^TM^ LC-18 column (5 μm particle size; Sigma-Aldrich) with UV detection at 254 nm (Jasco, UV-2075 Plus, Japan). Detailed methodology can be found elsewhere (see [30]). The repeatability of the sub-sample measured twice was very high (ICC = 0.97, 95% CI = 0.911–0.989, *F*_14,15_ = 62.2, *P* < 0.001). Recently, it was found that plasma triglyceride and MDA levels might correlate at least in certain species [35]. Although we did not measure triglycerides from the samples of the present study conducted in 2011–2012, we did so in a recent study conducted in 2014–2015 and found no association between triglycerides and MDA in house sparrows (*β* (SE) = –0.13 (0.15), *F*_1,58_ = 0.82, *P* = 0.370, *R*^2^ = 0.01).

### Condition, immunity measures and coccidian infestation

Packed red blood cell volume or hematocrit (Ht %) determines the oxygen transportation capacity of the peripheral blood, and thus it is frequently used as a proxy of condition in wild birds, where low Ht % values can be interpreted as signs of a poor condition [36]. Ht % was expressed as the proportion of the erythrocyte fraction to the total amount of blood measured after the capillaries were centrifuged (with digital caliper, ± 0.01 mm). Ht % was not measured for samples collected at *t*_0_, but was quantified shortly prior to this (4 and 8 November), hence we used the latter to test whether Ht % differed between the experimental groups before the treatment.

The humoral components of innate immunity (i.e., the levels of the natural antibodies and complement) were assessed using a modified hemolysis–hemagglutination assay [37], described in detail elsewhere [38]. The only modification related to our previous work was that here we used a freshly isolated 2% Wistar red blood cell suspension. Briefly, the blood was collected into a heparinized blood collection tube, centrifuged at 300 g for 10 min at 4°C and then washed three times in PBS (1:1, v:v). In this assay, agglutination reflects the activity of the natural antibodies (NAbs), while lysis represents the interaction between the natural antibodies and the complement [37,39].

Coccidians are unicellular epithelial parasites that are amongst the most pervasive parasites of birds [40]. At the end of the experiment, sparrows were placed in individual cages to quantify the level of *Isospora lacazei* coccidian infestation by measuring the rate of oocyst shedding two consecutive days (for details, see [41,42]). Coccidians from the genus *Isospora* shed oocysts predominantly during the late afternoons, therefore fecal samples were collected shortly before sunset. The number of oocysts was counted in McMaster chambers and the infestation intensity was expressed as the number of oocysts per g of feces. The oocyst counts of the two days were averaged for each individual and this value was used in the statistical analyses.

### Statistical procedures

Birds were housed in two separate aviaries, therefore we used mixed-effects models with aviary identity included in all models as a random factor. When analyzing variables that were measured repeatedly during the experiment (baseline CORT, body mass, Ht% and redox state variables), bird ID was entered in the models as an additional random factor. For Gaussian response variables (body mass, Ht %, all four oxidative physiology markers and coccidian infestation) we used general linear mixed-effects models (LMEs; ‘lme’ function of the R package ‘nlme’; [43]). For innate immunity variables we used generalized linear mixed-effects models (GLMMs; ‘glmer’ function of the R package ‘lme4’; [44]) with Poisson error distribution. We assessed the effect of population of origin by comparing models with and without population as a fixed factor and using likelihood ratio statistics in the case of each response variable. Including population of origin did not increase the models’ fit, therefore birds originating from the two populations were pooled throughout the subsequent analyses. All models included two fixed factors, treatment groups and sampling sessions, and also their interaction. In addition, we added to these models body mass as a continuous covariate and its interaction with treatment to assess whether CORT implantation has a condition-dependent effect on physiology. The control group was set as reference group throughout the analyses. Model summary tables were called by functions ‘summary’ or ‘Anova’ (the latter from R package ‘car’; [45]), which use type III SS. The former yields estimates (*β*) and *t*- or *z*-statistics (LME and GLMM, respectively), while the latter gives *χ*^2^ statistics. We also assessed the effect of treatment on response traits for each sampling session in separation to check whether treatment has an effect only during some of the sessions. All statistical analyses were carried out as implemented in R computing environment version 3.2.0 [46]. All the tests were two-tailed and *P* ≤ 0.05 was considered significant throughout. Model assumptions were assessed by graphical diagnosis.

### Ethics statement

Birds were handled in strict accordance with good animal welfare and ethical prescriptions. The sparrows were housed in indoor aviaries enriched with perches and nest boxes (for hiding and roosting), had *ad libitum* access to a mixture of seeds (sunflower, wheat, barley and corn) that was supplemented with grated boiled eggs every 4th day, and they received fresh drinking water every day. Grit was also provided for helping seed digestion and dust bathing. Aviary circumstances are described in detail elsewhere [47]. Birds seemingly habituated to captive conditions during the three months of acclimation between early August and middle of November, since after the initial weight loss during the first week of captivity they regained weight by the start of the experiment (mean (g) ± SE; field: 30.0 ± 0.28; first week in captivity: 27.0 ± 0.22; after acclimation and before CORT implantation: 29.5 ± 0.32). Two birds died for unknown reasons during the almost six months of the study. Note, however, that the survival rates of wild and other aviary populations are seldom higher [48]. The rest of the sparrows were released in good condition at the site of capture on 26 January 2012. Experimentation was approved by the Romanian Academy of Sciences (permission #2257) and the study complied with the laws of Romania.

## Results

Experimental groups did not differ in body mass, oxidative physiology and immunity (all *P* ≥ 0.108) prior the treatment (i.e. *t*_0_). Note, however, that Ht % was lower in the control group a few days before *t*_0_ (mean ± SE for control vs. CORT-treated: 55.77 ± 0.74 vs. 58.85 ± 0.89; *F*_1,35_ = 7.17, *P* = 0.011).

### Treatment effectiveness

**Fig 1:**
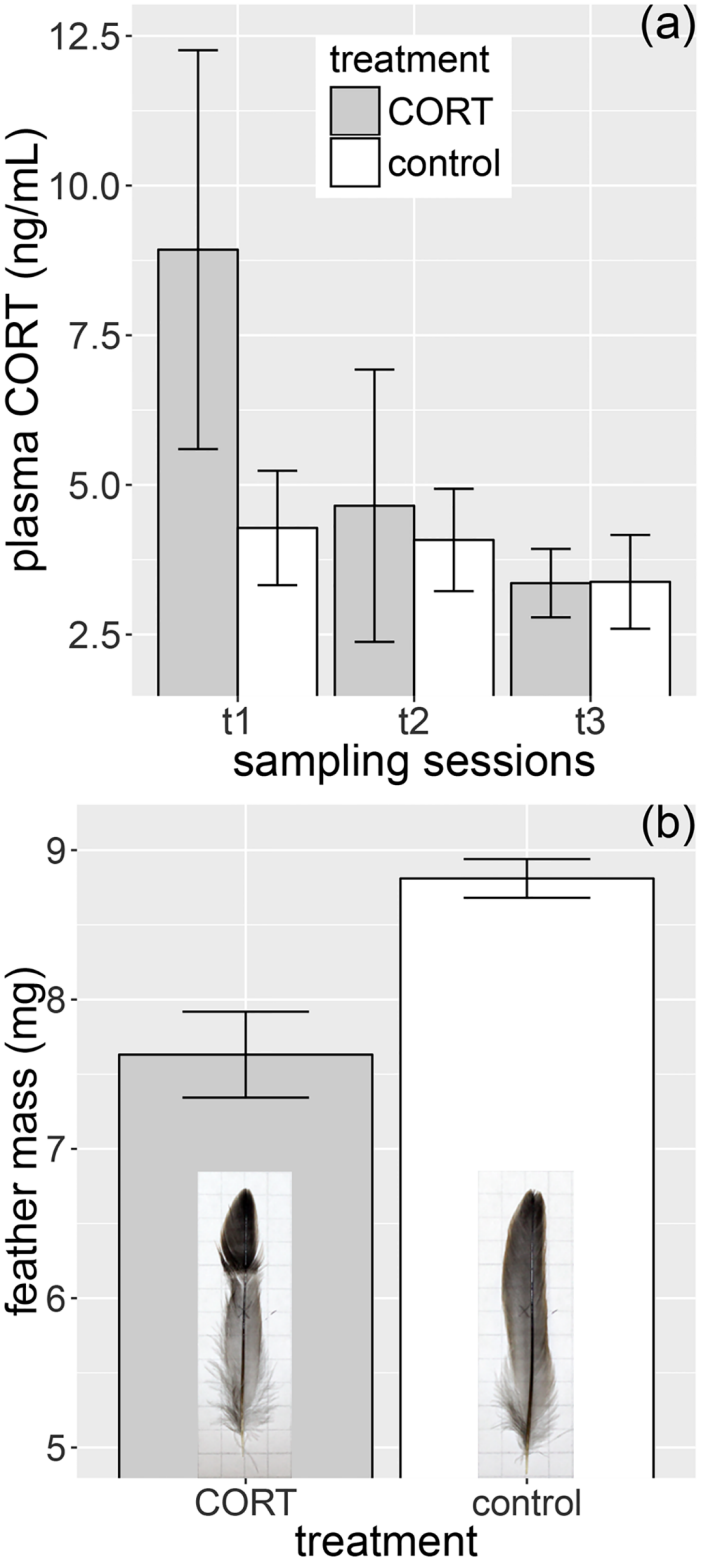
Treatment effectiveness. The effects of CORT-implantation on (a) plasma CORT levels during the first, second and third post-treatment sampling sessions (*t*_1_, *t*_2_ and *t*_3_, respectively) and (b) feather mass of replacement middle rectrices in post-molting house sparrows (feather insets are representative for the two treatment groups). Mean ± 1 SE are shown throughout. Sample sizes for CORT levels are 11 at *t*_1_, 13 at *t*_2_ and 12 at *t*_3_.

Based on the small subset of sparrows that were sampled for baseline CORT, we found that the pellet implants significantly increased the hormone level of CORT-treated birds (LME, *χ*^2^_1_ = 4.33, *P* = 0.037), but hormone levels did not differ between sampling sessions (LME, *χ*^2^_1_ = 0.19, *P* = 0.909) and the treatment × sampling session interaction was also non-significant (*χ*^2^_2_ = 2.78, *P* = 0.249). There was a marginally non-significant difference between treatment groups during *t*_1_ (*β* (SE) = 4.67 (2.24), *t* = 2.08, *P* = 0.059), while no difference was observed between treatment groups during *t*_2_ and *t*_3_ (*t*_2_: *β* (SE) = 0.64 (1.99), *t* = 0.32, *P* = 0.755; *t*_3_: *β* (SE) = –0.03 (2.07), *t* = 0.02, *P* = 0.987; Fig 1a).

The regrown retrices in CORT-treated birds had substantially smaller mass than in control birds (mean masses for control and CORT-treated groups are 8.81 and 7.63 mg, respectively; LME, *χ*^2^_1_ = 17.29, *P* < 0.001; Fig 1b). The effect of CORT-treatment on feather synthesis was also reflected by the aberrant coloration at basal parts of replacement feathers in CORT-treated birds (see feather insets on Fig 1b).

### Condition and physiology

**Fig 2:**
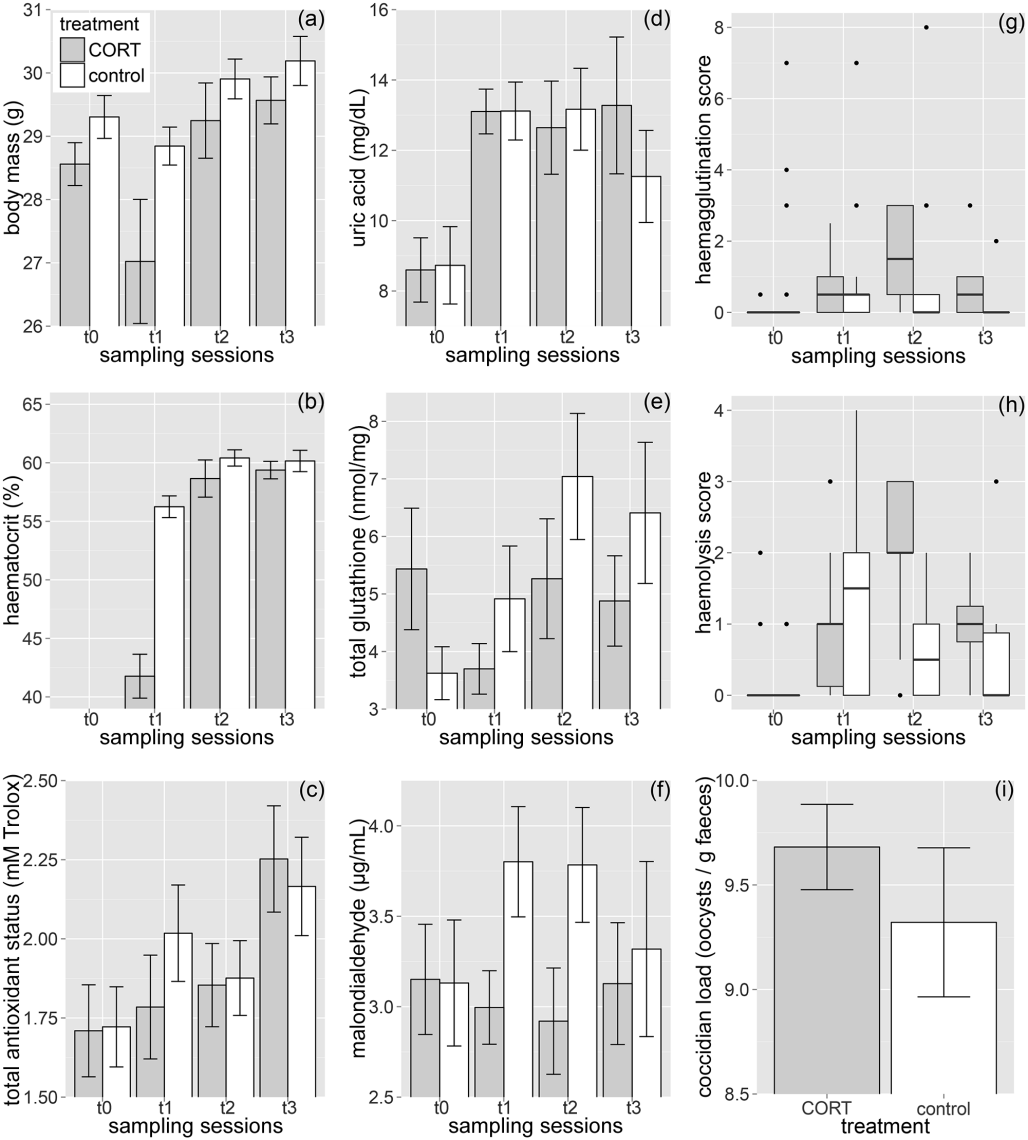
Physiological effects of chronic CORT treatment. The effects of CORT-implantation on (a) body mass, (b) hematocrit, (c) total antioxidant status, (d) uric acid, (e) total glutathione, (f) malondialdehyde, (g) hemagglutination, (h) hemolysis and (i) coccidian infestation in post-molting house sparrows. Sampling sessions: *t*_0_ –pre-treatment, *t*_1_, *t*_2_ and *t*_3_ –first, second and third post-treatment, respectively. Hematocrit was not measured during the pre-treatment sampling session (but see Materials and methods and Results), while coccidian load was only counted after the termination of the experiment. Mean ± 1 SE are shown throughout, except for panels (g) and (h) where median, inter-quartile range and range are plotted, and dots denote outliers. Body mass and hematocrit was measured for all birds at *t*_1_, *t*_2_ and *t*_3_, while samples sizes for oxidative stress and immunity variables are 27 at *t*_1_, 26 at *t*_2_ and 26 at *t*_3_.

Similarly to the effects of treatment on baseline CORT titers, implants had transient and mild effects on condition and physiology. CORT-treated birds had lower body mass and significantly lower Ht % values (Table 1). This treatment effect is apparent at *t*_1_ (body mass: *χ*^2^_1_ = 5.13, *P* = 0.024; Ht %: *χ*^2^_1_ = 55.44, *P* < 0.001), but both of these differences disappeared by *t*_2_ and *t*_3_ (body mass: all *P* > 0.248; Ht %: all *P* > 0.258; Fig 2a and 2b). Note that Ht % was lower in CORT-treated than control birds at *t*_1_, despite the opposite trend prior to the experiment (see above).

**Table 1:** Physiological effects of chronic CORT treatment. LMEs for body mass, hematocrit (Ht %), redox state markers (TAS—total antioxidant status, UA—uric acid, tGSH—total glutathione, MDA—malondialdehyde; for residual TAS, see Methods) and coccidian infestation, and GLMMs for innate immunity variables (natural antibodies [NAbs] by hemagglutination score and complement by hemolysis scores). Degrees of freedom are 1 for treatment and 3 for sampling and treatment × sampling throughout. Significant effects are marked in boldface and marginally significant effects (0.05 < *P* ≤ 0.10) in italic.

Response	Treatment (T)		Sampling (S)		T × S	
	*χ*^2^	*P*	*χ*^2^	*P*	*χ*^2^	*P*
**Body mass**	1.30	0.254	12.10	**0.007**	6.02	0.111
**Ht %**	84.45	**< 0.001**	13.54	**0.001**	69.28	**< 0.001**
**TAS**	0.01	0.942	5.99	0.112	1.22	0.748
**Residual TAS**	0.00	0.962	4.69	0.196	0.97	0.809
**UA**	0.01	0.924	17.98	**< 0.001**	1.51	0.681
**tGSH**	2.75	*0*.*097*	10.31	**0.016**	6.57	*0*.*087*
**MDA**	0.00	0.961	5.86	0.118	4.62	0.202
**Coccidiosis**	0.73	0.394	–	–	–	–
**NAbs**	4.01	0.260	15.12	**0.010**	2.96	0.398
**Complement**	2.63	0.105	25.64	**< 0.001**	7.05	*0*.*070*

CORT treatment did not affect TAS, UA and tGSH levels (Table 1; Fig 2c–2e) at any of the post-treatment sampling sessions (TAS: all *P* > 0.292; UA: all *P* > 0.272; tGSH: all *P* > 0.242). Oxidative damage to lipids (i.e. MDA) appeared to be also similar between experimental groups (Table 1). Nonetheless, CORT-treated birds had significantly lower MDA levels than control birds both at *t*_1_ and *t*_2_, while this difference decreased and was not significant at *t*_3_ (*t*_1_: *χ*^2^_1_ = 4.88, *P* = 0.027; *t*_2_: *χ*^2^_1_ = 3.99, *P* = 0.046; *t*_3_: *P* = 0.813; Fig 2f).

CORT treatment did not influence the activity of NAbs (i.e. hemagglutination score), nor did it have an effect on the complement system (i.e. hemolysis score; Table 1). The activity of NAbs was similar between the two experimental groups at all three post-treatment sampling sessions (all *P* > 0.136; Fig 2g), while CORT-treated birds had significantly higher complement scores at *t*_2_ and marginally higher at *t*_3_ (*t*_1_: *χ*^2^_1_ = 0.50, *P* = 0.481; *t*_2_: *χ*^2^_1_ = 8.54, *P* = 0.004; *t*_3_: *χ*^2^_1_ = 2.87, *P* = 0.090; Fig 2h). The level of infestation by coccidians was similar between the two experimental groups following *t*_3_ (Table 1; Fig 2i).

The addition of body mass to the above models had an effect only on innate immunity. Leaner birds had lower NAb levels (GLMM, *β* (SE) = 0.97 (0.29), *z* = 3.33, *P* < 0.001) and lower complement activity (GLMM, *β* (SE) = 0.67 (0.34), *z* = 2.01, *P* = 0.045). In case of NAbs, the interaction between body mass and CORT treatment was also significant (GLMM, *β* (SE) = –1.09 (0.28), *z* = 3.83, *P* < 0.001). The latter interaction indicated that the activity of NAbs was unrelated to body mass in the CORT-treated group (*β* (SE) = 0.09 (0.06), *z* = 1.50, *P* = 0.133), but was positively correlated with body mass in the control group (*β* (SE) = 1.27 (0.32), *z* = 4.02, *P* < 0.001). In case of the complement, inclusion of body mass did not modify the effect of CORT treatment (comparison of treatment groups per sampling session, *t*_1_: *P* = 0.990; *t*_2_: *P* = 0.009; *t*_3_: *P* = 0.227).

## Discussion

In this study we experimentally induced an elevation of circulating CORT levels within the natural baseline range in house sparrows and we investigated several response variables at the (1) cellular and (2) individual (physiological) levels. At these levels, we found that CORT-treated birds had (1) lower amount of oxidative damage, but similar glutathione concentrations, (2) an initially lowered hematocrit, but on a longer term an enhanced activity of their innate immune system, and (3) temporarily lower body mass, but similar coccidian infestation. These results suggest that small increases in circulating CORT levels may have complex effects on condition and physiology, as the direction, strength and duration of CORT-induced effects varied largely among response variables.

### Treatment effectiveness

CORT pellet implantation transiently increased plasma CORT titers within the range of natural variation in baseline CORT of house sparrows characteristic to the post-molt–wintering life-history stages (~8.5 ng/mL), not reaching the acute stress-induced levels (above 15–20 ng/mL) [49]. It should be noted, however, that—in order to avoid capture stress-induced interference with CORT treatment—we did not sample birds during the first days post-implantation, when a spike in CORT titer that exceeds the baseline range might have occurred [6,50]. Moreover, CORT treatment reduced the mass of the newly-grown tail feathers, which corroborates a well-documented negative effect of elevated CORT levels on feather development [28,51–53]. Together, these results clearly indicate that the exogenous hormone implantation significantly increased circulating CORT levels, at least during the first few days following implantation, but the effective duration of the treatment was shorter than anticipated. The number of birds that were sampled within three minutes and were therefore suitable for baseline CORT measurements was small, reducing the power of statistical analyses of this parameter. Nonetheless, the transient nature of the CORT treatment is probably not merely due to small sample size. During the years that passed since we conducted this experiment, it became clear that time-release pellets often do not provide the expected constant release dynamics in birds. Such pellets often produce an initial peak in hormone levels and then the hormone content rapidly depletes [6,54–59].

### Condition

Weight loss is one of the most consistent physiological effects of CORT (e.g. [60]; reviewed by [27,61]; but see [50]). Our results corroborate these findings, indicating that shortly after the implantation CORT-treated birds were lighter in weight than control birds. A direct inhibitory effect of GCs on erythropoiesis has also been demonstrated ([27,36,50]; but see [60]). Our result are in concert with this view, as the packed red blood cell volume (i.e. hematocrit) also dropped in CORT-treated, but not in control birds. These transient adverse effects of CORT treatment on condition might be indicative that the pellets produce an initial spike in hormone release that exceeds the baseline range of CORT titer (see e.g. [6] for 7-days release pellets), despite pellets being developed for steady hormone release. Alternatively, the priming of oxidative physiological and immunological systems by a chronic stress state (see below) might have costs that manifest as weight loss, erythropoiesis deficiency and lower quality feathers that all might have negative fitness consequences [53,62]. Our experiment also highlights that these effects of CORT are also apparent in the energetically least demanding period of the annual cycle (post-molt) and under *ad libitum* diet conditions. The latter further suggests that CORT implants produced an initial peak in CORT levels that affected body condition in a transient manner.

### Innate immunity and coccidiosis

The effects of GCs on immunity are often portrayed as inhibitory [12,61,63,64]. Nonetheless, whether GCs suppress, redistribute or enhance the immune capacity highly depends on the form, duration and intensity of stress response and the immune measure under scrutiny [11,12,64]. This complexity might explain the controversial results that concern the immunomodulatory effect of GCs [5,12,61,64]. Recent studies suggest that components of the innate immune system can be enhanced during short-term stress exposure ([12]; see also [65]). Moreover, innate immune responses were found to be the less sensitive to nutritional stress [11]. In line with these notions, Stier et al. [7] showed that CORT pellet implantation reduced antibody production, but did not affect constitutive innate immunity in barn owl *Tyto alba* nestlings. Bourgeon & Raclot [66] implanted female eiders *Somateria mollissima* with CORT pellets and found that treatment suppressed antibody production, but had no effect on cutaneous immunity, which involves innate and acquired cellular immune components. Butler et al. [67] found that CORT-implanted American kestrel *Falco sparverius* nestlings had higher level of cutaneous immune response than sham-implanted nestlings. Our results corroborate these three studies by demonstrating a positive effect of CORT-implantation on the activity of the complement system. Similarly to our findings, dietary restriction, that is known to double baseline GC levels in rats [68], facilitates adaptive immune response [69].

One obvious cost of impaired immune function during stressful conditions could be the increased susceptibility to parasites and pathogens. Previous studies showed both positive [24] and negative association [11] between the level of coccidian infestation and that of GCs. In our experiment the level of infestation by coccidians did not differ between the two treatment groups at the end of the experiment. Although coccidians are combated by cell-mediated acquired immunity via CD4+ helper T-cells [24], we previously showed that coccidian infestation can be associated with higher scores of natural antibodies and complement in house sparrows ([42]; but see [70]). Nonetheless, treatment group differences in the activity of the complement was only apparent shortly after the implantation and not at later sampling occasions. Therefore, the lack of difference in the level of coccidiosis between the two treatment groups is in line with the transient effect of CORT-implantation on immunity, which might both have disappeared by the time coccidian infestation was assessed (i.e. end of the experiment).

### Redox state

Contrary to the expected adverse effects of chronic stress on oxidative homeostasis [17], recent studies showed also positive effects. For instance, baseline cortisol levels are positively associated with non-enzymatic antioxidant capacity in alpine marmosets *Marmota marmota* [71], and long-term exogenous cortisol treatment increased GSH levels [72]. Angelier et al. [73] also developed a framework that proposes oxidative stress as a mediator of the linkage between stress and telomere dynamics, and this framework involves GSH as a major component. Our findings do not corroborate the abovementioned results, as non-enzymatic antioxidant levels were similar in the two treatment groups. On the other hand though, the level of peroxidative lipid damage (MDA) remained low in the CORT-treated birds and increased in the control birds over the course of the treatment period. Male house sparrows experience an increased oxidative damage both in their natural environment [74] and in captivity [70] during the post-molt period. Our result thus suggests that CORT-treated birds were better able to cope with this period in terms of oxidative stress resistance, while control birds experienced significantly higher lipid peroxidation. At least two mechanisms can explain this result. First, GCs can remold the lipid composition of membranes, by means of reducing the fraction of polyunsaturated fatty acids, and ultimately membrane vulnerability to peroxidation [68]. Second, GCs induce heat shock proteins that are involved in cell damage repair [75]. These mechanisms, either in separation or in synergy, might have prevented the increase of MDA in CORT-treated birds.

These results are consistent with the concept of hormesis, i.e. an improvement in stress resistance (here against oxidation) at low increase of CORT levels, but a decline of the same parameters at high increase of CORT levels [4]. Studies that employed high intensity GC treatments found increased DNA damage and elevated peroxidation of lipids, but when GCs were administered at the short term (few hours to 4–5 days) antioxidant defenses were upregulated (reviewed by [76]). For instance, in a field study on wild barn owl nestlings, CORT pellet implantation induced a five-fold increase in baseline CORT and resulted in a subsequent lower resistance to oxidative challenge relative to controls [7]. However, smaller increases in GC levels reduced oxidative damage in rats and broiler chickens [21,68], while heat stress increased the concentration of antioxidant enzymes in chickens [20,21,77]. Dietary restriction in rats that produced an increase in CORT levels comparable to the magnitude of our treatment also resulted in mitigated lipid peroxidation and activated heat shock protein response [68,78]. Our results are also reminiscent of CORT’s non-linear effect on spatial memory and a related brain region, the hippocampus. A low experimental increase of CORT in a food-caching species, the mountain chickadees *Poecile gambeli*, increased spatial memory and did not affect hippocampal volume and cells contrary to high experimental increase of CORT [79,80].

## Conclusions

The physiological end-point of elevated baseline CORT levels is contingent upon magnitude and duration of increase in baseline CORT. Our treatment increased CORT levels within the baseline range over a short time period and, as such, it probably primed the physiological setting points and evoked shifts within the limits of controllable processes. Such adjustments generally help to maintain homeostasis or to cope with mild and predictable stressors contrary to an uncontrollable (‘runaway’) situation (i.e. long-term high CORT release and inefficient negative feed-back of the HPA axis; [81]). Collectively, these might explain the generally weak and, when significant, positive cellular and molecular physiological consequences of CORT pellet implantation in our study. However, this physiological priming might be paid off by weight loss, deficiency in erythropoiesis and low feather quality, which are costs that can have negative fitness consequences [53,62].

## References

[1] Hau M, Casagrande S, Ouyang JQ, Baugh AT. Glucocorticoid-mediated phenotypes in vertebrates: multilevel variation and evolution. Adv Study Behav. 2016;48: 41–115. doi: 10.1016/bs.asb.2016.01.002

[2] Crespi EJ, Williams TD, Jessop TS, Delehanty B. Life history and the ecology of stress: how do glucocorticoid hormones influence life-history variation in animals? Funct Ecol. 2013;27: 93–106. doi: 10.1111/1365-2435.12009

[3] Romero LM, Dickens MJ, Cyr NE. The reactive scope model—A new model integrating homeostasis, allostasis, and stress. Horm Behav. 2009;55: 375–389. doi: 10.1016/j.yhbeh.2008.12.009 19470371 10.1016/j.yhbeh.2008.12.009

[4] Costantini D, Metcalfe NB, Monaghan P. Ecological processes in a hormetic framework. Ecol Lett. 2010;13: 1435–1447. doi: 10.1111/j.1461-0248.2010.01531.x 20849442 10.1111/j.1461-0248.2010.01531.x

[5] Merrill L, Angelier F, O’Loghlen AL, Rothstein SI, Wingfield JC. Sex-specific variation in brown-headed cowbird immunity following acute stress: a mechanistic approach. Oecologia. 2012;170: 25–38. doi: 10.1007/s00442-012-2281-4 22382434 10.1007/s00442-012-2281-4

[6] Müller C, Almasi B, Roulin A, Breuner CW, Jenni-Eiermann S, Jenni L. Effects of corticosterone pellets on baseline and stress-induced corticosterone and corticosteroid-binding-globulin. Gen Comp Endocrinol. 2009;160: 59–66. doi: 10.1016/j.ygcen.2008.10.018 18996387 10.1016/j.ygcen.2008.10.018

[7] Stier KS, Almasi B, Gasparini J, Piault R, Roulin A, Jenni L. Effects of corticosterone on innate and humoral immune functions and oxidative stress in barn owl nestlings. J Exp Biol. 2009;212: 2085–2091. doi: 10.1242/jeb.024406 19525435 10.1242/jeb.024406

[8] Almasi B, Roulin A, Korner-Nievergelt F, Jenni-Eiermann S, Jenni L. Coloration signals the ability to cope with elevated stress hormones: effects of corticosterone on growth of barn owls are associated with melanism. J Evol Biol. 2012;25: 1189–1199. doi: 10.1111/j.1420-9101.2012.02508.x 22530630 10.1111/j.1420-9101.2012.02508.x

[9] Crossin GT, Love OP, Cooke SJ, Williams TD. Glucocorticoid manipulations in free-living animals: considerations of dose delivery, life-history context and reproductive state. Funct Ecol. 2016;30: 116–125. doi: 10.1111/1365-2435.12482

[10] Cohen AA, Martin LB, Wingfield JC, McWilliams SR, Dunne JA. Physiological regulatory networks: ecological roles and evolutionary constraints. Trends Ecol Evol. 2012;27: 428–435. doi: 10.1016/j.tree.2012.04.008 22613457 10.1016/j.tree.2012.04.008

[11] Apanius V. Stress and immune defense. Adv Study Behav. 1998;27: 133–153.

[12] Martin LB. Stress and immunity in wild vertebrates: timing is everything. Gen Comp Endocrinol. 2009;163: 70–76. doi: 10.1016/j.ygcen.2009.03.008 19318107 10.1016/j.ygcen.2009.03.008

[13] McEwen BS, Wingfield JC. The concept of allostasis in biology and biomedicine. Horm Behav. 2003;43: 2–15. doi: 10.1016/S0018-506X(02)00024-7 12614627 10.1016/s0018-506x(02)00024-7

[14] Dhabhar FS, McEwen BS. Enhancing versus suppressive effects of stress hormones on skin immune function. Proc Natl Acad Sci USA. 1999;96: 1059–1064. doi: 10.1073/pnas.96.3.1059 9927693 10.1073/pnas.96.3.1059 PMC15350

[15] Sapolsky RM, Romero LM, Munck AU. How do glucocorticoids influence stress responses? Integrating permissive, suppressive, stimulatory, and preparative actions. Endocr Rev. 2000;21: 55–89. doi: 10.1210/edrv.21.1.0389 10696570 10.1210/edrv.21.1.0389

[16] Halliwell B, Gutteridge JMC. Free radicals in biology and medicine. 4th ed Oxford, UK: Oxford University Press; 2007.

[17] Costantini D, Marasco V, Møller AP. A meta-analysis of glucocorticoids as modulators of oxidative stress in vertebrates. J Comp Physiol B. 2011;181: 447–456. doi: 10.1007/s00360-011-0566-2 21416253 10.1007/s00360-011-0566-2

[18] Costantini D, Møller AP. Does immune response cause oxidative stress in birds? A meta-analysis. Comp Biochem Physiol A. 2009;153: 339–344. doi: 10.1016/j.cbpa.2009.03.010 19303455 10.1016/j.cbpa.2009.03.010

[19] Spiers JG, Chen H-JC, Sernia C, Lavidis NA. Activation of the hypothalamic-pituitary-adrenal stress axis induces cellular oxidative stress. Neuroendocr Sci. 2015;8: 456 doi: 10.3389/fnins.2014.00456 25646076 10.3389/fnins.2014.00456 PMC4298223

[20] Lin H, Decuypere E, Buyse J. Oxidative stress induced by corticosterone administration in broiler chickens (*Gallus gallus domesticus*) 1. Chronic exposure. Comp Biochem Physiol B. 2004;139: 737–744. doi: 10.1016/j.cbpc.2004.09.013 15581806 10.1016/j.cbpc.2004.09.013

[21] Lin H, Decuypere E, Buyse J. Oxidative stress induced by corticosterone administration in broiler chickens (*Gallus gallus domesticus*) 2. Short-term effect. Comp Biochem Physiol B. 2004;139: 745–751. doi: 10.1016/j.cbpc.2004.09.014 15581807 10.1016/j.cbpc.2004.09.014

[22] You J-M, Yun S-J, Nam KN, Kang C, Won R, Lee EH. Mechanism of glucocorticoid-induced oxidative stress in rat hippocampal slice cultures. Can J Physiol Pharmacol. 2009;87: 440–447. doi: 10.1139/y09-027 19526038 10.1139/y09-027

[23] Speakman JR, Blount JD, Bronikowski AM, Buffenstein R, Isaksson C, Kirkwood TBL, et al Oxidative stress and life histories: unresolved issues and current needs. Ecol Evol. 2015;5: 5745–5757. doi: 10.1002/ece3.1790 26811750 10.1002/ece3.1790 PMC4717350

[24] Sild E, Meitern R, Männiste M, Karu U, Hõrak P. High feather corticosterone indicates better coccidian infection resistance in greenfinches. Gen Comp Endocrinol. 2014;204: 203–210. doi: 10.1016/j.ygcen.2014.05.026 24953456 10.1016/j.ygcen.2014.05.026

[25] Romero LM, Platts SH, Schoech SJ, Wada H, Crespi E, Martin LB, et al Understanding stress in the healthy animal—potential paths for progress. Stress. 2015;18: 491–497. doi: 10.3109/10253890.2015.1073255 26365223 10.3109/10253890.2015.1073255

[26] Landys MM, Ramenofsky M, Wingfield JC. Actions of glucocorticoids at a seasonal baseline as compared to stress-related levels in the regulation of periodic life processes. Gen Comp Endocrinol. 2006;148: 132–149. doi: 10.1016/j.ygcen.2006.02.013 16624311 10.1016/j.ygcen.2006.02.013

[27] Breuner CW, Delehanty B, Boonstra R. Evaluating stress in natural populations of vertebrates: total CORT is not good enough. Funct Ecol. 2013;27: 24–36. doi: 10.1111/1365-2435.12016

[28] Jovani R, Rohwer S. Fault bars in bird feathers: mechanisms, and ecological and evolutionary causes and consequences. Biol Rev. 2017;92: 1113–1127. doi: 10.1111/brv.12273 27062218 10.1111/brv.12273

[29] Lendvai ÁZ, Bókony V, Chastel O. Coping with novelty and stress in free-living house sparrows. J Exp Biol. 2011;214: 821–828. doi: 10.1242/jeb.047712 21307069 10.1242/jeb.047712

[30] Bókony V, Lendvai ÁZ, Vágási CI, Pătraș L, Pap PL, Németh J, et al Necessity or capacity? Physiological state predicts problem-solving performance in house sparrows. Behav Ecol. 2014;25: 124–135. doi: 10.1093/beheco/art094

[31] Cohen A, Klasing K, Ricklefs R. Measuring circulating antioxidants in wild birds. Comp Biochem Physiol B. 2007;147: 110–121. doi: 10.1016/j.cbpb.2006.12.015 17303461 10.1016/j.cbpb.2006.12.015

[32] Galván I, Alonso-Alvarez C. An intracellular antioxidant determines the expression of a melanin-based signal in a bird. PLoS One. 2008;3: e3335 doi: 10.1371/journal.pone.0003335 18833330 10.1371/journal.pone.0003335 PMC2556083

[33] Hõrak P, Sild E, Soomets U, Sepp T, Kilk K. Oxidative stress and information content of black and yellow plumage coloration: an experiment with greenfinches. J Exp Biol. 2010;213: 2225–2233. doi: 10.1242/jeb.042085 20543121 10.1242/jeb.042085

[34] Del Rio D, Stewart AJ, Pellegrini N. A review of recent studies on malondialdehyde as toxic molecule and biological marker of oxidative stress. Nutr Metab Cardiovasc Dis. 2005;15: 316–328. doi: 10.1016/j.numecd.2005.05.003 16054557 10.1016/j.numecd.2005.05.003

[35] Pérez-Rodríguez L, Romero-Haro AA, Sternalski A, Muriel J, Mougeot F, Gil D, et al Measuring oxidative stress: the confounding effect of lipid concentration in measures of lipid peroxidation. Physiol Biochem Zool. 2015;88: 345–351. doi: 10.1086/680688 25860832 10.1086/680688

[36] Fair J, Whitaker S, Pearson B. Sources of variation in haematocrit in birds. Ibis. 2007;149: 535–552. doi: 10.1111/j.1474-919X.2007.00680.x

[37] Matson KD, Ricklefs RE, Klasing KC. A hemolysis–hemagglutination assay for characterizing constitutive innate humoral immunity in wild and domestic birds. Dev Comp Immunol. 2005;29: 275–286. doi: 10.1016/j.dci.2004.07.006 15572075 10.1016/j.dci.2004.07.006

[38] Pap PL, Czirják GÁ, Vágási CI, Barta Z, Hasselquist D. Sexual dimorphism in immune function changes during the annual cycle in house sparrows. Naturwissenschaften. 2010;97: 891–901. doi: 10.1007/s00114-010-0706-7 20706704 10.1007/s00114-010-0706-7

[39] Buehler DM, Piersma T, Matson K, Tieleman BI. Seasonal redistribution of immune function in a migrant shorebird: annual-cycle effects override adjustments to thermal regime. Am Nat. 2008;172: 783–796. doi: 10.1086/592865 18999941 10.1086/592865

[40] Greiner EC. *Isospora*, *Atoxoplasma*, and *Sarcocystis* In: Atkinson CT, Thomas NJ, Hunter DB, editors. Parasitic Diseases of Wild Birds. New York, NY: Wiley-Blackwell; 2008 pp. 108–119.

[41] Pap PL, Vágási CI, Czirják GÁ, Titilincu A, Pintea A, Barta Z. Carotenoids modulate the effect of coccidian infection on the condition and immune response in moulting house sparrows. J Exp Biol. 2009;212: 3228–3235. doi: 10.1242/jeb.031948 19801427 10.1242/jeb.031948

[42] Pap PL, Vágási CI, Czirják GÁ, Titilincu A, Pintea A, Osváth G, et al The effect of coccidians on the condition and immune profile of molting house sparrows (*Passer domesticus*). Auk. 2011;128: 330–339. doi: 10.1525/auk.2011.10142

[43] Pinheiro J, Bates D, DebRoy S, Sarkar D, R Core Team. nlme: Linear and Nonlinear Mixed Effects Models. R package version 3.1–120. http://CRAN.R-project.org/package=nlme; 2015.

[44] Bates D, Maechler M, Bolker B, Walker S. lme4: Linear mixed-effects models using Eigen and S4. R package version 1.1–7. http://CRAN.R-project.org/package=lme4; 2014.

[45] Fox J, Weisberg S. An R Companion to Applied Regression. 2nd ed http://socserv.socsci.mcmaster.ca/jfox/Books/Companion; 2011.

[46] R Core Team. R: a language and environment for statistical computing. Vienna, Austria http://www.R-project.org/: R Foundation for Statistical Computing; 2015.

[47] Vágási CI, Pap PL, Barta Z. Haste makes waste: accelerated molt adversely affects the expression of melanin-based and depigmented plumage ornaments in house sparrows. PLoS One. 2010;5: e14215 doi: 10.1371/journal.pone.0014215 21151981 10.1371/journal.pone.0014215 PMC2997061

[48] Liker A, Bókony V. Larger groups are more successful in innovative problem solving in house sparrows. Proc Natl Acad Sci USA. 2009;106: 7893–7898. doi: 10.1073/pnas.0900042106 19416834 10.1073/pnas.0900042106 PMC2683070

[49] Romero LM, Cyr NE, Romero RC. Corticosterone responses change seasonally in free-living house sparrows (*Passer domesticus*). Gen Comp Endocrinol. 2006;149: 58–65. doi: 10.1016/j.ygcen.2006.05.004 16774754 10.1016/j.ygcen.2006.05.004

[50] Beck ML, Davies S, Moore IT, Schoenle LA, Kerman K, Vernasco BJ, et al Beeswax corticosterone implants produce long-term elevation of plasma corticosterone and influence condition. Gen Comp Endocrinol. 2016;233: 109–114. doi: 10.1016/j.ygcen.2016.05.021 27222349 10.1016/j.ygcen.2016.05.021

[51] Romero LM, Strochlic D, Wingfield JC. Corticosterone inhibits feather growth: potential mechanism explaining seasonal down regulation of corticosterone during molt. Comp Biochem Physiol A. 2005;142: 65–73. doi: 10.1016/j.cbpa.2005.07.014 16125989 10.1016/j.cbpa.2005.07.014

[52] DesRochers DW, Reed JM, Awerman J, Kluge JA, Wilkinson J, van Griethuijsen LI, et al Exogenous and endogenous corticosterone alter feather quality. Comp Biochem Physiol A. 2009;152: 46–52. doi: 10.1016/j.cbpa.2008.08.034 18804171 10.1016/j.cbpa.2008.08.034

[53] Jenni-Eiermann S, Helfenstein F, Vallat A, Glauser G, Jenni L. Corticosterone: effects on feather quality and deposition into feathers. Methods Ecol Evol. 2015;6: 237–246. doi: 10.1111/2041-210X.12314

[54] Bonier F, Martin PR, Wingfield JC. Maternal corticosteroids influence primary offspring sex ratio in a free-ranging passerine bird. Behav Ecol. 2007;18: 1045–1050. doi: 10.1093/beheco/arm075

[55] Henriksen R, Groothuis TG, Rettenbacher S. Elevated plasma corticosterone decreases yolk testosterone and progesterone in chickens: linking maternal stress and hormone-mediated maternal effects. PLoS One. 2011;6: e23824 doi: 10.1371/journal.pone.0023824 21886826 10.1371/journal.pone.0023824 PMC3160319

[56] Almasi B, Roulin A, Jenni L. Corticosterone shifts reproductive behaviour towards self-maintenance in the barn owl and is linked to melanin-based coloration in females. Horm Behav. 2013;64: 161–171. doi: 10.1016/j.yhbeh.2013.03.001 23583559 10.1016/j.yhbeh.2013.03.001

[57] Quispe R, Trappschuh M, Gahr M, Goymann W. Towards more physiological manipulations of hormones in field studies: comparing the release dynamics of three kinds of testosterone implants, silastic tubing, time-release pellets and beeswax. Gen Comp Endocrinol. 2015;212: 100–105. doi: 10.1016/j.ygcen.2015.01.007 25623144 10.1016/j.ygcen.2015.01.007

[58] Tartu S, Bustamante P, Angelier F, Lendvai ÁZ, Moe B, Blévin P, et al Mercury exposure, stress and prolactin secretion in an Arctic seabird: an experimental study. Funct Ecol. 2016;30: 596–604. doi: 10.1111/1365-2435.12534

[59] Hennin HL, Wells-Berlin AM, Love OP. Baseline glucocorticoids are drivers of body mass gain in a diving seabird. Ecol Evol. 2016;6: 1702–1711. doi: 10.1002/ece3.1999 26925215 10.1002/ece3.1999 PMC4755010

[60] Grace JK, Froud L, Meillère A, Angelier F. House sparrows mitigate growth effects of post-natal glucocorticoid exposure at the expense of longevity. Gen Comp Endocrinol. 2017;253: 1–12. doi: 10.1016/j.ygcen.2017.08.011 28811198 10.1016/j.ygcen.2017.08.011

[61] Roberts ML, Buchanan KL, Hasselquist D, Evans MR. Effects of testosterone and corticosterone on immunocompetence in the zebra finch. Horm Behav. 2007;51: 126–134. doi: 10.1016/j.yhbeh.2006.09.004 17049519 10.1016/j.yhbeh.2006.09.004

[62] Bowers EK, Hodges CJ, Forsman AM, Vogel LA, Masters BS, Johnson BGP, et al Neonatal body condition, immune responsiveness, and hematocrit predict longevity in a wild bird population. Ecology. 2014;95: 3027–3034. doi: 10.1890/14-0418.1 25505800 10.1890/14-0418.1 PMC4260523

[63] Martin LB, Gilliam J, Han P, Lee K, Wikelski M. Corticosterone suppresses cutaneous immune function in temperate but not tropical house sparrows, *Passer domesticus*. Gen Comp Endocrinol. 2005;140: 126–135. doi: 10.1016/j.ygcen.2004.10.010 15613275 10.1016/j.ygcen.2004.10.010

[64] Braude S, Tang-Martinez Z, Taylor GT. Stress, testosterone, and the immunoredistribution hypothesis. Behav Ecol. 1999;10: 345–350. doi: 10.1093/beheco/10.3.345

[65] Sapolsky RM. The influence of social hierarchy on primate health. Science. 2005;308: 648–652. doi: 10.1126/science.1106477 15860617 10.1126/science.1106477

[66] Bourgeon S, Raclot T. Corticosterone selectively decreases humoral immunity in female eiders during incubation. J Exp Biol. 2006;209: 4957–4965. doi: 10.1242/jeb.02610 17142684 10.1242/jeb.02610

[67] Butler MW, Leppert LL, Dufty AM Jr.. Effects of small increases in corticosterone levels on morphology, immune function, and feather development. Physiol Biochem Zool. 2010;83: 78–86. doi: 10.1086/648483 19929638 10.1086/648483

[68] Caro P, Gómez J, Sanz A, Portero-Otín M, Pamplona R, Barja G. Effect of graded corticosterone treatment on aging-related markers of oxidative stress in rat liver mitochondria. Biogerontology. 2007;8: 1–11. doi: 10.1007/s10522-006-9026-x 16823605 10.1007/s10522-006-9026-x

[69] Speakman JR, Mitchell SE. Caloric restriction. Mol Aspects Med. 2011;32: 159–221. doi: 10.1016/j.mam.2011.07.001 21840335 10.1016/j.mam.2011.07.001

[70] Pap PL, Sesarman A, Vágási CI, Buehler DM, Pătraş L, Versteegh M a., et al No evidence for parasitism-linked changes in immune function or oxidative physiology over the annual cycle of an avian species. Physiol Biochem Zool. 2014;87: 729–739. doi: 10.1086/676934 25244384 10.1086/676934

[71] Costantini D, Ferrari C, Pasquaretta C, Cavallone E, Carere C, von Hardenberg A, et al Interplay between plasma oxidative status, cortisol and coping styles in wild alpine marmots, *Marmota marmota*. J Exp Biol. 2012;215: 374–383. doi: 10.1242/jeb.062034 22189781 10.1242/jeb.062034

[72] Birnie-Gauvin K, Peiman KS, Larsen MH, Aarestrup K, Willmore WG, Cooke SJ. Short-term and long-term effects of transient exogenous cortisol manipulation on oxidative stress in juvenile brown trout. J Exp Biol. 2017;220: 1693–1700. doi: 10.1242/jeb.155465 28209806 10.1242/jeb.155465

[73] Angelier F, Costantini D, Blévin P, Chastel O. Do glucocorticoids mediate the link between environmental conditions and telomere dynamics in wild vertebrates? A review. Gen Comp Endocrinol. 2017; doi: 10.1016/j.ygcen.2017.07.007 28705731 10.1016/j.ygcen.2017.07.007

[74] Pap PL, Pătraş L, Osváth G, Buehler DM, Versteegh MA, Sesarman A, et al Seasonal patterns and relationships among coccidian infestations, measures of oxidative physiology, and immune function in free-living house sparrows over an annual cycle. Physiol Biochem Zool. 2015;88: 395–405. doi: 10.1086/681243 26052636 10.1086/681243

[75] Currie S, LeBlanc S, Watters MA, Gilmour KM. Agonistic encounters and cellular angst: social interactions induce heat shock proteins in juvenile salmonid fish. Proc R Soc B. 2010;277: 905–913. doi: 10.1098/rspb.2009.1562 19923129 10.1098/rspb.2009.1562 PMC2842717

[76] Costantini D. On the measurement of circulating antioxidant capacity and the nightmare of uric acid. Methods Ecol Evol. 2011;2: 321–325. doi: 10.1111/j.2041-210X.2010.00080.x

[77] Altan Ö, Pabuçcuoğlu A, Altan A, Konyalioğlu S, Bayraktar H. Effect of heat stress on oxidative stress, lipid peroxidation and some stress parameters in broilers. Br Poult Sci. 2003;44: 545–550. doi: 10.1080/00071660310001618334 14584844 10.1080/00071660310001618334

[78] Yu BP, Chung HY. Stress resistance by caloric restriction for longevity. Ann N Y Acad Sci. 2006;928: 39–47. doi: 10.1111/j.1749-6632.2001.tb05633.x 10.1111/j.1749-6632.2001.tb05633.x 11795526

[79] Pravosudov V V. Long-term moderate elevation of corticosterone facilitates avian food-caching behaviour and enhances spatial memory. Proc R Soc B. 2003;270: 2599–2604. doi: 10.1098/rspb.2003.2551 14728783 10.1098/rspb.2003.2551 PMC1691552

[80] Pravosudov V V., Omanska A. Prolonged moderate elevation of corticosterone does not affect hippocampal anatomy or cell proliferation rates in mountain chickadees (*Poecile gambeli*). J Neurobiol. 2005;62: 82–91. doi: 10.1002/neu.20069 15389682 10.1002/neu.20069

[81] Hõrak P, Cohen A. How to measure oxidative stress in an ecological context: methodological and statistical issues. Funct Ecol. 2010;24: 960–970. doi: 10.1111/j.1365-2435.2010.01755.x

